# The impact of the three-level collaboration exercise on collaboration and leadership during scenario-based hospital evacuation exercises using flexible surge capacity concept: a mixed method cross-sectional study

**DOI:** 10.1186/s12913-023-09882-x

**Published:** 2023-08-14

**Authors:** Phatthranit Phattharapornjaroen, Eric Carlström, Pongsakorn Atiksawedparit, Lina Dahlén Holmqvist, Dhanesh Pitidhammabhorn, Yuwares Sittichanbuncha, Amir Khorram-Manesh

**Affiliations:** 1 Institute of Clinical Sciences, Department of Surgery, Sahlgrenska Academy, University of Gothenburg, Gothenburg, 40530 Sweden; 2 Department of Emergency Medicine, Faculty of Medicine Ramathibodi Hospital, Mahidol University, Bangkok, 10400 Thailand; 3 Institute of Health and Care Sciences, Sahlgrenska Academy, University of Gothenburg, Gothenburg, 40100 Sweden; 4 Gothenburg Emergency Medicine Research Group, Sahlgrenska Academy, University of Gothenburg, Gothenburg, 40530 Sweden; 5 USN School of Business, University of South-Eastern Norway, Kongsberg, 3603 Norway; 6 Faculty of Medicine Ramathibodi Hospital, Chakri Naruebodindra Medical Institute, Mahidol University, Samut Prakan, 10540 Thailand; 7 Institute of Medicine, Department of Internal Medicine and Clinical Nutrition, Sahlgrenska University Hospital, Gothenburg, 40530 Sweden; 8 Disaster Medicine Center, Sahlgrenska Academy, University of Gothenburg, Gothenburg, 40530 Sweden

**Keywords:** Flexible surge capacity, Preparedness, Hospital evacuations, 3-level collaboration exercise, Disaster partnership, Leadership

## Abstract

**Background:**

Hospitals play a crucial role in responding to disasters and public health emergencies. However, they are also vulnerable to threats such as fire or flooding and can fail to respond or evacuate adequately due to unpreparedness and lack of evacuation measures. The United Nations Office for Disaster Risk Reduction has emphasised the importance of partnerships and capacity building in disaster response. One effective way to improve and develop disaster response is through exercises that focus on collaboration and leadership. This study aimed to examine the effectiveness of using the 3-level collaboration (3LC) exercise in developing collaboration and leadership in districts in Thailand, using the concept of flexible surge capacity (FSC) and its collaborative tool during a hospital evacuation simulation.

**Methods:**

A mixed-method cross-sectional study was conducted with 40 participants recruited from disaster-response organisations and communities. The data from several scenario-based simulations were collected according to the collaborative elements (Command and control, Safety, Communication, Assessment, Triage, Treatment, Transport), in the disaster response education, “Major Incident Medical Management and Support” using self-evaluation survey pre- and post-exercises, and direct observation.

**Results:**

The 3LC exercise effectively facilitated participants to gain a mutual understanding of collaboration, leadership, and individual and organisational flexibility. The exercise also identified gaps in communication and the utilisation of available resources. Additionally, the importance of early community engagement was highlighted to build up a flexible surge capacity during hospital evacuation preparedness.

**Conclusions:**

the 3LC exercise is valuable for improving leadership skills and multiagency collaboration by incorporating the collaborative factors of Flexible Surge Capacity concept in hospital evacuation preparedness.

**Supplementary Information:**

The online version contains supplementary material available at 10.1186/s12913-023-09882-x.

## Background

Disasters caused by natural and man-made hazards and public health emergencies (DPHEs) may potentially overwhelm healthcare systems indirectly or target hospitals directly, resulting in a surge of patients, strain on staff, equipment, and hospital spaces, and even enforce hospital evacuation [1, 2]. Given hospitals’ critical role throughout the disaster cycle, particularly in the response phase, their inadequate preparedness and vulnerability can lead to profound socioeconomic losses, fatalities, and increased suffering [3–9]. Recognising the significance of the issue, The United Nations Office for Disaster Risk Reduction (UNDRR) has recently emphasised the importance of multiagency partnerships in enhancing disaster response capabilities and capacity building, particularly when incidents expand, and planned resources are insufficient or cannot be delivered. These partnerships involve collaboration within healthcare organizations and with external entities, including public and private entities, non-governmental organisations, and communities [10]. Achieving effective collaboration, which entails shared goals, requires multiagency communication to coordinate available resources and facilitate cooperation in various areas. Each new event is based on new circumstances, which require swift adaptations of the leadership [11] as well as being able to involve skills from collaborating organisations [12]. However, progress in these partnerships has been surprisingly slow, with limited levels of communication, cooperation, or coordination, particularly in the context of hospital evacuations, which are intricate procedures demanding careful measures and resources [3]. One reason for this may be a lack of preparedness because of insufficient exercise. Exercises are often outlined as drills, focusing on actions within respective organisations rather than exercises that are carried out in collaboration between organisations. Drills are based on mechanistic logic and aim to train a professional skill where the participant performs his/her specialised trade but are not really integrated with participants from other organisations [13, 14]. Moreover, collaboration has an organic focus aiming to integrate experience and skills from different organisations and get participants to collaborate in order to handle a complex event [15, 16]. Hospital evacuations are regarded as extremely complex operations that may become necessary in cases where the hospital’s functionality is compromised due to internal events, such as fire, technical issues threatening the lives of patients, and explosions. Furthermore, certain hospitals situated in high-risk areas contend with frequent major natural incidents, leading to partial or total evacuations [17–19]. In these situations, quick, reliable, and evidence-based decisions should be made, which may be challenging to leadership, demanding comprehensive situation assessment and surge planning to ensure the safe transport of patients and staff to secure and compatible locations and appropriate treatment [20–23].

The COVID-19 pandemic has served as a crucial turning point in healthcare services, exposing the insufficiencies in healthcare surge capacity and leadership capability, highlighting the urgency of multiagency collaboration among healthcare organisations and across public and private sectors. Moreover, evidence from previous studies shows that hospital staff often lack preparedness due to limited knowledge of disaster response principles and inadequate awareness of contingency plans [4, 18, 19, 24, 25]. During the COVID-19 pandemic, some hospitals faced contamination, necessitating patient evacuations, leading to substantial resource consumption and revealing their lack of preparedness [26, 27]. In response to these challenges, several hospitals have improvised and actively engaged local communities. They used community’s unhampered spaces and facilities to serve as alternative care facilities and enlisted community volunteers in patients’ health screening and monitoring [26, 28]. This collaborative approach, known as Flexible Surge Capacity (FSC), allows for scalable support from the community to healthcare systems during major incidents. Previous studies have explored the feasibility and effectiveness of FSC from theoretical and practical perspectives, indicating its potential applicability beyond the pandemics [29–32]. Implementing FSC could prove indispensable in various DPHEs’ scenarios; hence fostering and refining multiagency collaboration, using FSC protocols is vital to preparedness during hospital evacuations. Therefore, as previously reported, educational initiatives, including simulation training, ranging from tabletop exercise to full-scale simulation with a focus on collaborative techniques, should become integrals to disaster preparedness, as they have been proven to enhance knowledge and response capability in emergencies [33–35]. Thailand is a country that frequently experiences disasters [36], for example, flooding that affected numerous hospitals in the central region in 2011, the 2004 tsunami that damaged structures along the southern coast of Thailand, and the COVID-19 pandemic that contaminated hospitals [4, 37]. Using the collaborative elements of Command and control, Safety, Communication, Assessment, Triage, Treatment, and Transport (CSCATTT), a recent review of Thai hospitals’ preparedness identified several shortcomings in each of these elements. Among the critical issues are insufficiencies in leadership, communication, mutual assessment abilities, triage, and transport [24].

Since multiagency collaboration is significant in responses to hospital evacuation events, the three Level Collaboration (3LC), which focuses on training disaster management collaboration can favorably be used. 3LC exercise emphasizes collaboration between the main principal partners in an emergency: the police department, fire services, and prehospital staff. The exercise consist of 2–3 scenarios requiring all partners to work together, highlighting overlapping responsibilities and addressing asymmetries. Seminars following each scenario enable discussion of gaps, shortcomings, actions, reactions, and suggestions for alternative strategies [35]. Previous studies have demonstrated significant associations between collaboration, learning, and usefulness in the context of the 3LC exercise [35, 38]. By practising collaboration with other entities (experiencing the limitations and capabilities of others) and using collaborative tools, participants gain new knowledge (learning) that can be effectively applied in practice, enhancing their skills’ practicality (usefulness) [34, 35, 38]. However, most studies have focused on partnerships either between hospitals and their entities or blue-light organizations, lacking a broader examination of collaborations across organizations, nations, private actors, and communities [35].

.

Given the potential for hospital threats that might require evacuations such as flooding, fire or explosion, and the insufficient preparedness of the hospitals, partnerships and collaborations in DPHEs, [4, 19, 24, 39], this study focused on developing collaboration using the FSC concept in response to hospital evacuation. To the best of our knowledge, this study is the first to assess collaboration and leadership among healthcare systems and external entities through simulation training which may offer an alternative way to explore and prepare hospitals for all aspects of surge capacity, including staff, stuff, space, and systems. Therefore, this study aimed to assess the effectiveness of using the 3LC exercise in developing collaboration and leadership in districts in Thailand, using the FSC concept and its collaborative tool during hospital evacuation scenarios.

## Method

### Study setting and design

This mixed-method cross-sectional study employed a combination of participants’ self-evaluation, direct observations by exercise instructors, and video records during a two-day 3LC simulation exercise. The exercise was conducted at the Chakri Naruebodindra Medical Institute, Faculty of Medicine Ramathibodi Hospital, with the hospital serving as the centre for disaster impacts and responses and the surrounding districts as the study’s context. The selection of the districts was motivated by their strategic geographical locations, which exhibit an approximately equal distribution between agriculture and industrial sectors. These areas also face recurrent flooding incidents within a city with a substantial population of more than 1.4 million inhabitants [40], and their proximity to Bangkok International Airport. Consequently, four of the six districts in Samut Prakan province, including Mueang Samut Prakan, Bang Bo, Bang Phli, and Bang Sao Thong, were purposively selected based on considerations of geographical suitability, risk assessments, and the feasibility of collaboration to partake in this exercise (i.e. FSC) [35].

Participants were divided into groups of 7–8 individuals with diverse roles and responsibilities to allow for heterogeneity within the groups while maintaining homogeneity among them. The number of participants in each group supported a sense of sharing and enabled discussion [41, 42]. The self-evaluation form in a native language, provided in Supplementary Material 1, was distributed to participants both before and immediately after the exercise. This form allowed participants to assess their own performance and provide insights into their experiences. During the exercise, the 3LC exercise instructors closely observed the collaborative and leadership elements exhibited by the participants. Their observations were guided by the observational checklist prepared by the research team, available in Supplementary Material 2. The instructors possessed a 3LC exercise education and had a minimum of 5 years of experience in disaster response or had actively participated in at least one hospital’s disaster preparedness exercises. To ensure data accuracy, all exercise sessions and seminars were recorded and then transcribed verbatim. These recordings served as supporting data for self-evaluation and observation.

### Study participants

The organisations included hospitals and provincial public health organisations (healthcare authority) affiliated with the Ministry of Health, provincial administration, city municipals, and the Department of Disaster Prevention and Mitigation (DDPM), which included Fire departments affiliated with the Ministry of Interior, police departments affiliated with the Royal Thai Police, and community facilities such as religious institutes, schools, local clinics, hotels, etc.

The official invitation letters, research details, and researchers’ contact information were sent to the chiefs of each organisation through postal mail. Each organisation was asked to purposively select 2–4 representatives based on the following inclusion criteria: they should (1) know the organisation’s capacity and capability and (2) be responsible for communicating the need for future collaborations to the organisational management committees. The research details were provided to representatives again before consent was obtained to ensure they understood the voluntary nature of their participation.

### The 3LC exercise

This study’s activity comprised 3 rounds of disaster response functional exercises and 3 rounds of seminars following each training session [35]. During the functional exercises, participants were presented with hospital evacuation scenarios that required them to engage in discussions and devise solutions using resources available within their respective organisations while maintaining their actual roles. These scenarios were designed to enable participants to practice and demonstrate various forms of collaboration within the context of disaster response. Following their responses to the scenarios, participants engaged in seminars. The seminars included 2 open-ended questions: (1) what they did during the response to the disaster scenario exercise, and (2) what they could change in their actions based on the experience gained to improve their response in a subsequent similar scenario. These questions aimed to improve collaboration, learning and usefulness by encouraging self-evaluation, critiques of individual or group activities, and the exchange of suggestions for alternative strategies. These seminars played a pivotal role in fostering a deeper understanding of collaboration dynamics and leadership qualities, thus contributing to the overall assessment of collaboration and leadership and group dynamic development during the two-day 3LC exercise. The exercise instructors did not interfere in the ongoing discussions when the teams collaborated by collectively identifying challenges and creatively discussing solutions. In situations of over-polite, hesitant or inactive engagement from the participants, the exercise instructors encouraged the teams to collaborate by being focused on the task, opening up, giving their views, elaborating plans and operating them.

### Scenarios

Exercise scenarios (Supplementary Material 3) were obtained by experienced disaster and emergency medicine experts based on their knowledge, previous literature review, experiences from similar events, and the 3-year accumulated data of internal documents on vulnerability and hazard assessments in the area, which reported the fire, flooding, and infectious disease as leading threats. The dynamic components of the scenarios were based on the pragmatic paradigm of disaster preparedness exercise and the complexities of the healthcare system [32, 35, 43, 44].

Using a nominal group technique [45], four representatives from hospitals’ hazard vulnerability committees (2 physicians, 1 nurse, and 1 paramedic) with more than 5 years of experience in the field were recruited to discuss and ensure that scenarios were relevant to the community and hospital contexts [27, 32, 36, 46–48]. The Nominal Group Technique is akin to focus groups but with more structure and an immediate, quantitative output.

### Study materials

The evaluation of participants’ performance and perspectives during the functional exercise were collected using an evaluation tool. The tool was adapted from the aforementioned CSCATTT acronym in the Major Incident Medical Management and Support (MIMMS) educational courses [48–53]. Furthermore, the tool was integrated with the FSC concept, developed by three emergency and disaster medicine experts using a nominal group technique (2 physicians and 1 nurse) [47]. The evaluation process involved 2 components as follows:

- Self-evaluation form consisted of a Likert scale assessing the participant’s knowledge of the collaborative elements (CSCATTT), ranging from unknown (0) to literacy and can convey knowledge (5). In addition to the Likert scale, the form included open-ended questions that aimed to capture participants’ perspectives on the FSC concept. These questions allowed participants to provide more detailed insights and reflections. (Supplementary Material 1)
- Observational checklist comprised 2 parts. The first part recorded participants’ performance during the functional exercise, indicating whether they performed specific actions or not. Additionally, a rubric scale was utilized to evaluate the levels of performance. The second part of the checklist included free written comment forms for observers to provide any additional comments or insights they deemed necessary (Supplementary Material 2).

### Data processing and analysis

The data collected through self-evaluation forms and observation were entered into Microsoft Office Excel version 16.71 for initial processing and then analysed using Stata version 17. Descriptive statistics were used to present the data in counts, proportions, medians, and interquartile ranges. The Wilcoxon-signed rank test was used to compare pre-and post-self-evaluation on the Likert scale. The Likert scale was further categorised into three collaborative levels (poor (scales 0,1), fair (scales 2,3), and good (scales 4,5)). Differences in levels between pre- and post-exercise were analysed by the Chi-square test or Fisher’s exact test (if the expected number in each cell was below 5) of the proportion of poor, fair, and good ratings.

For the qualitative analysis, the answers from self-evaluation open-ended questions and observation notes were compared with data obtained from self-criticism during seminars. A deductive approach inspired by Graneheim and Lundman was employed for qualitative content analysis [54, 55]. The data were read several times to gain a comprehensive understanding and then divided into meaningful units. These units were further condensed, abstracted, and coded. The coded data were subsequently grouped based on similarities and differences. Lastly, reflection on the themes, a review of the literature related to the themes, and all authors’ discussions provided relevancy to sorted and unified coded into themes of collaborative elements (CSCATTT).

## Results

To assess the impacts of the 3LC exercise on collaboration and leadership development, self-evaluation and performances of participants from disaster-response organisations in selected districts were explored during the exercise. Fifty participants were initially recruited, with 40 remaining until the end of the study. The age of participants ranged from 23 to 58 years, with 74% working on the front line of various organisations (e.g., hospitals and provincial administration/city municipals). Overall, 58% were hospital representatives from 4 different hospitals in the Samut Prakan area. The characteristics of the participants are presented in Table 1.

**Table 1:** General characteristics of participants

	Overall (n = 50)	Group 1 (n = 8)	Group 2 (n = 6)	Group 3 (n = 7)	Group 4 (n = 7)	Group 5 (n = 7)	Group 6 (n = 8)	Group 7 (n = 7)
**Gender**								
Male, n (%)	23 (46.0)	2 (25.0)	2 (33.3)	3 (42.9)	5 (71.4)	3 (42.9)	3 (37.5)	5 (71.4)
Age (Median, IQR*)	35.5 (27.0,45.0)	31 (26.3,36.5)	36.5 (33.6,55.0)	33 (25.0,41.0)	47 (44.0,54.0)	32 (26.0,44.0)	32 (27.5,48.8)	38 (30.0,54.0)
**Level of operation, n (%)**								
Frontliner	37 (74.0)	4 (50)	4 (66.7)	5 (71.5)	6 (85.7)	6 (85.7)	7 (87.5)	5 (71.5)
Middle-level manager	12 (24.0)	4 (50)	1 (16.7)	2 (28.5)	1 (14.3)	1 (14.3)	1 (12.5)	2 (28.5)
High-level manager	1 (2.0)	-	1 (16.7)	-	-	-	-	-
**Organization, n (%)**								
Hospitals	29 (58.0)	4 (50.0)	3 (50.0)	4 (57.1)	3 (42.9)	4 (57.1)	6 (75.0)	5 (71.4)
Provincial Public Health	4 (8.0)	1 (12.5)	1 (16.7)	1 (14.3)		1 (14.3)	1 (12.5)	-
Police Department	4 (8.0)	-	-	-	1 (14.3)	1 (14.3)	1 (12.5)	-
Provincial Administration/ City municipals	6 (12.0)	1 (12.5)	1 (16.7)	2 (28.6)	1 (14.3)	1 (14.3)	-	-
Department of Disaster Prevention and Mitigation (including fire department)	3 (6.0)	1 (12.5)	1 (16.7)	-	1 (14.3)	-	-	-
**Community, n (%)**								
Religious place	2 (4.0)	1 (12.5)	-	-	-	-	-	1 (14.3)
School	1 (2.0)	-	-	-	1 (14.3)	-	-	
Non-governmental organization	1 (2.0)	-	-	-	-	-	-	1 (14.3)

*IQR = Interquartile rage, Groups represented participating groups during the 3LC exercise

### Self-evaluation

The 3LC exercise significantly improved participants’ perceived levels of collaboration (Table 2; *P* < 0.001). Significant improvements in the proportions of collaboration (i.e., poor, fair, and good) were found in almost all areas between pre- and post-exercise, particularly *command and control* (*P* = 0.001) and *hospital evacuation* elements (*P* < 0.001). In contrast, no differences were observed for *treatment* (*P* = 0.085) and *transport* (*P* = 0.060).

**Table 2:** Comparison of collaboration levels regarding disaster response systems and hospital evacuation between pre- and post-exercise

Collaborative elements (n = 40)	Collaboration	Pre-exercise n (%)	Post-exercise n (%)	P-value**
Command and control	Poor	10 (24.4)	1 (2.5)	0.001
	Fair	27 (65.9)	16 (40.0)	
	Good	4 (9.8)	23 (57.5)	
	Median (IQR)	2 (1.5,3.0)	4 (3,4.5)	-5.481* < 0.001*
Safety	Poor	8 (20.0)	2 (5.0)	0.017
	Fair	28 (**70**.0)	13 (32.5)	
	Good	4 (10.0)	25 (62.5)	
	Median (IQR)	2 (2.0,3.0)	4 (3,5)	-5.343* < 0.001*
Communication	Poor	10 (25.0)	0	0.013
	Fair	26 (65.0)	17 (42.5)	
	Good	4 (10.0)	23 (57.5)	
	Median (IQR)	2 (1.5,3.0)	4 (3,4.5)	-5.452* < 0.001*
Assessment	Poor	14 (35.0)	2 (5.0)	0.034
	Fair	21 (52.5)	13 (32.5)	
	Good	5 (12.5)	25 (62.5)	
	Median (IQR)	2 (1.0,3.0)	4 (3,4.5)	-5.523* < 0.001*
Triage	Poor	10 (25.0)	2 (5.0)	0.037
	Fair	26 (65.0)	12 (30.0)	
	Good	4 (10.0)	26 (65.0)	
	Median (IQR)	2 (1.5,3.0)	4 (3.0,4.5)	-5.373* < 0.001*
Treatment	Poor	15 (37.5)	2 (5.0)	0.085
	Fair	20 (50.0)	14 (35.0)	
	Good	5 (12.5)	24 (60.0)	
	Median (IQR)	2 (1.0,2.0)	4 (3.0,4.0)	-5.351* < 0.001*
Transport	Poor	15 (37.5)	2 (5.0)	0.060
	Fair	19 (47.5)	15 (37.5)	
	Good	6 (15.0)	23 (57.5)	
	Median (IQR)	2 (1.0,2.0)	4 (3.0,4.5)	-5.423* < 0.001*
Hospital evacuation	Poor	15 (37.5)	2 (5.0)	0.000
	Fair	22 (55.0)	16 (40.0)	
	Good	3 (7.5)	22 (55.0)	
	Median (IQR)	2 (1.0,3.0)	4 (3.0,4.0)	-5.568* < 0.001*

Each item was rated on a Likert scale from 0 (unknown) to 5 (literacy and ability to convey knowledge). *Differences between pre- and post-self-evaluation exercises were analyzed by the Wilcoxon sign rank test. **Differences in collaboration between pre- and post-exercise were analyzed by Chi-square test or Fisher’s exact test (if the expected number in each cell was below 5) of the proportion of poor (0,1), fair (2,3) and good (4,5) ratings

### Qualitative data from self-evaluation and observation

**Fig. 1:**
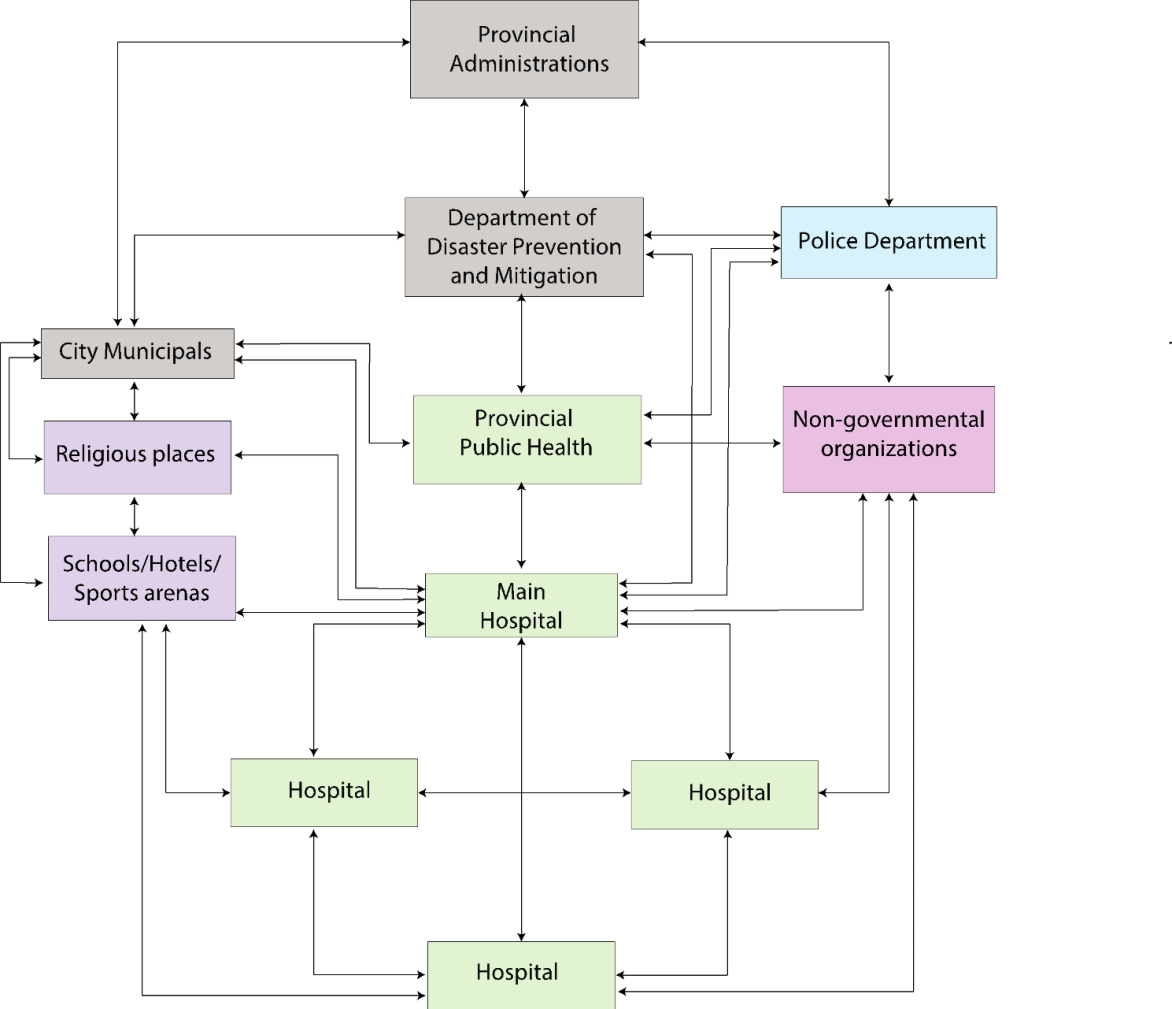
Communications and coordination among multi-agency organisations during the 3LC exercise

The 3LC exercise allowed participants to collaborate in all elements of disaster response (CSCATTT). Observed back-and-forth communications among organisations are illustrated in Fig. 1. The communications aimed to share information on resources, establish guidelines for cooperation and coordination, and share ideas on each organisation’s expected roles and responsibilities. Participants engaged in the exercises and communicated across organizations. Moreover, community facilities were observed to be involved in several response activities.

**Fig. 2:**
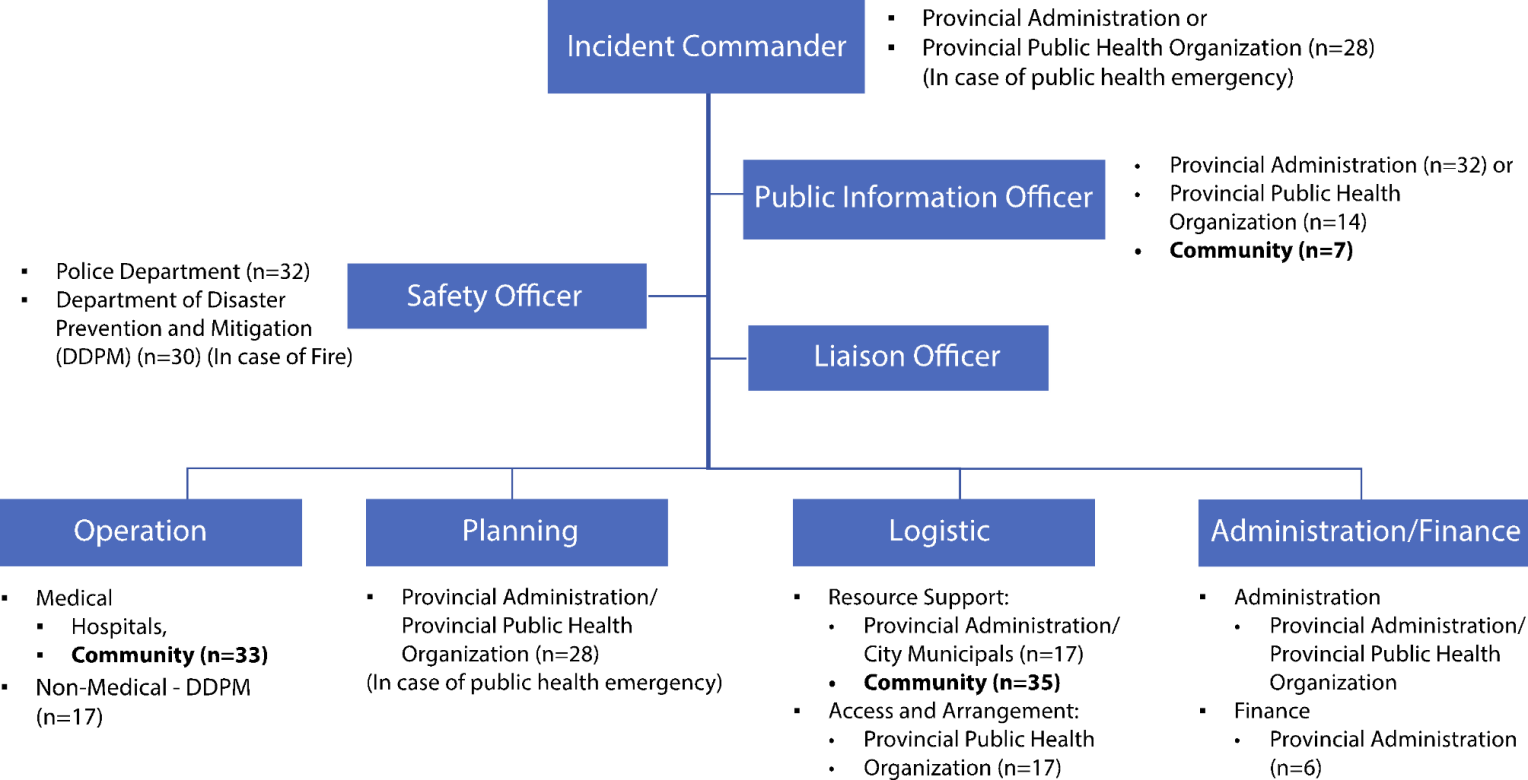
Perceived functions of each organisation in the Incident command system The data were concluded from the self-evaluation form and discussion during the 3LC exercise n is the frequency that the organisations were regarded to perform the role or have responsibilities to provide in the incident command system The community was bold in the figure to highlight its roles in the ICS

Additionally, data from self-evaluation open-ended questions demonstrated the perceived functions of each organisation in the incident command system are shown in Fig. 2. Most participants perceived the provincial administration and public health organisations as incident commanders and planning actors (n = 28), the community as contributors in medical operations (n = 33) and logistics parts (n = 35), and police officers as safety officers of events (n = 32) and organising traffic (n = 28). The representatives employed distinct leadership styles. When participants from the provincial administration took leadership roles, they exercised their central authorities and controlled response efforts. Conversely, participants from the healthcare system either endeavoured to cooperate and coordinate with others during response efforts, adapted their strategies to changing circumstances during responses or encouraged all members to participate in discussions.

Observational notes and transcribed recording data demonstrated participants’ leadership performances and styles (Table 3), and collaborative elements according to the CSCATTT (Table 4). In the *command-and-control* element, all groups designated leaders to act as incident commanders, either through selection or based on assigned roles. The leaders from the police department and provincial administration performed vertical commands, controls, and communication structures. As for leadership performance, different patterns emerged based on specific scenarios. During the pandemic scenario, hospital staff predominantly led the response. In contrast, when the scenario involved flooding affecting the entire district, representatives from the provincial administration played a more active role in leadership. In a scenario involving fire and explosion, the police officers took on incident management and leadership responsibilities. Most groups in this fire scenario used a consensus technique. In the *safety* elements, police officers and representatives from the DDPM demonstrated their responsibility for safety controls to maintain peace and order during incidents. Furthermore, the infectious control department was recruited to control the outbreak of infections, if necessary.

**Table 3:** Leadership performance and styles

Scenarios/ Organization	Group 1	Group 2	Group 3	Group 4	Group 5	Group 6	Group 7
Scenario 1: Flooding	Provincial public health	Provincial public health	Provincial administration	Hospitals	Hospitals	Hospitals	Non-governmental organization
Style	Passive	Active	Active	Consensus	Passive	Consensus	Active
Scenario 2: Pandemic	Hospitals	Provincial public health	Hospitals	Hospitals	Hospitals	Hospitals	Hospitals
Style	Consensus	Active	Consensus	Consensus	Passive	Active	Passive
Scenario 3: Fire and explosion	Hospitals	Provincial public health	Provincial administration	Hospitals	Police department	Hospitals	Hospitals
Style	Consensus	Passive	Passive	Consensus	Active	Consensus	Consensus

Data derived from observational notes and transcribed recording data

**Table 4:** Qualitative data from self-criticism and observations based on collaborative elements (CSCATTT)

Themes	Codes	Condensed and accumulated meaning units	Frequency
Command and control	Leaders were chosen	Leaders were chosen according to their organizational roles in the disaster plan (representatives from either healthcare authority or municipal authority) or have the original role in the middle to the high management level of the organizations.	26
	Leaders’ roles	Leaders acted as idea initiators rather than commanders and preferred horizontal communication for command and control (Healthcare professionals).	13
		Leaders acted as commanders and vertical commands were the primary communications. (Police departments, Provincial administration, and the Department of Disaster Prevention and Mitigation [DDPM])	4
	Responsibilities were assigned and controlled by the whole group	Tasks were distributed and assigned in accordance with the original roles of organizations and were controlled by all members.	23
	The incident command system was used.	The incident command system was proposed as the preferred managing system and used as a core element during responses.	6
	Extension of resources to community	The need for other organizations’ collaborations was a concern, particularly from community resources.	22
	Establish important areas.	Areas for decontamination, disease control/safe zone, treatments, and transportation routes were discussed and zoned.	20
Safety	Safety officer	Police officers or Fire fighters played the main roles in safety management to maintain peace and order.	18
	Safety management	Safe zones were established for disease control and transportation routes.	17
		The infectious team was mentioned for infectious control and personal protection for staff.	8
		Food and water sanitization during the incident was mentioned and planned	4
Communication	Internal communication	Internal communications were activated according to the disaster plan through voice announcements and social media platforms.	16
	Inter-organization communication	Inter-organization communications channels were initiated through online platforms or radio channels.	31
	Public communication	Public communication was established through the head of the community, community health volunteers, social media platforms that were easily accessed, and/or public announcements. Moreover, communications in disaster matters should concern benefits from crowdsourced information.	9
Assessment	Own resource evaluation	Organizations evaluated their own capacities and capabilities to provide healthcare and basic needs (food, water, place to stay) for staff, patients and families during response, and recovery.	26
	Surge planning	Other organizations involvement for mutual resources and supports	15
	Community engagement	Community resources were recruited into surge planning and response. Temple and mosque offered spaces for shelters or treatment areas, as well as food and water for survival supplies. Schools offered teachers and students to help with patients and equipment evacuation, as well as space for shelters or treatment areas.	4
Triage	Patient prioritization	Patients were prioritized and transported accordingly to appropriate areas.	19
Treatment	Treatment zone was set up	Patients were treated at appropriate areas which were either hospitals or field hospitals.	6
Transport	Patient transportation	Patient transportation were the primary discussion in all scenarios and all group. The common transportation channel was the usual referral system between hospitals.	16
	Device transportation	The device was moved with the patients, particularly in critical cases.	3
	Alternative means	The alternative transportation means that were recruited during the exercise were private cars from community, police cars, buses and boats from public and provincial units, and airplanes from military.	19
Opportunity for improvement	Command and control	Organize a well-structured command post	21
	Safety	Develop and implement safety policy, particularly hazardous issues, transportation traffic and access routes.	12
	Communication	Establish standard communication channels and information access points for staff and public.	9
	Assessment (Stuff)	Provide staff and patients’ survival supplies	5
	Transport (Stuff)	Develop practice guideline for stockpiling and logistics of medical device, and supplies.	9
	Staff	Provide educational initiatives for disaster preparedness for staff and people with disaster plan rehearsal	11
	Structure	Develop guideline for community areas’ utilization as shelters or treatment zones	6

In the *communication* element, participants demonstrated communications internally and inter-organizationally through normal referral channels and public announcements about the updated information on the situation were also discussed. However, social media sharing was a primary concern, and they recognised the lack of management and addressed the improvement opportunities in the areas. In the *assessment* element, participants conducted self-surge and surge planning for resource expansion during the disaster responses. Moreover, representatives from community places, e.g., schools and religious institutes, intensely engaged in supporting elements in surge capacity (i.e., staff, stuff, and space).

As *triage* and *treatment* element were specific to healthcare providers, they were solely performed by representatives from hospitals and provincial public health organisations. Lastly, regarding the *transport* element, all organisations’ participants shared their resources and logistics support. Representatives from DDPM, police departments, and provincial administrations presented their valuable resources, for example, boats, buses, cars, etc. Although these supplies were in good condition and ready to use, the process and regulations for recruiting these necessary resources still needed to be made available.

## Discussion

The 3LC exercise conducted in this study, using the concept of FSC and its tool, was found to improve collaboration between diverse entities and agencies, participants’ knowledge, and their understanding of collaborative elements. Since the 3LC technique includes time outs with space for short seminars, including metalevel reflections on what we did and could anything have been done differently, collaborative techniques were successively improved among the participants from the first to the third scenario.

Multi-agency organisations presented their capacities and capabilities (coordination). They agreed on the goals, shared resources, and shared responsibilities (cooperation), leading to leadership improvement opportunities, collaborations (sharing the same aims), and surge planning. This study was the first to involve community engagement in the first step of disaster preparedness, specifically in the case of a hospital evacuation. Moreover, this multi-agency collaboration aligns with the UNDRR’s strategic objectives to strengthen governance and catalyse actions of partnerships and stakeholders [10].

Due to the heterogeneity within the groups, management and leadership approaches were different across scenarios. It was evident that both organizational factors and the specific nature of the scenario influenced leadership performances. When representatives from hierarchical-based organizations, such as police departments and provincial administrations, assumed *command, control*, and *communication* roles, vertical measures with a directive approach were predominantly observed. This hierarchical structure has been successfully implemented in various incidents and deployed in several hospital incident command systems [24, 51, 53]. On the other hand, healthcare professionals took the lead in the scenario involving public health emergencies like a pandemic, and the organizational structure became predominantly horizontal, allowing for discussion [56, 57].

Various leadership styles were observed, including collaborative (consensus), which hospital representatives primarily used; adaptive (active), which representatives from provincial organizations predominantly employed; and participatory (passive), which was sporadically observed. These leadership approaches were consistent with the complexity of the healthcare system and cultures and the styles observed in a previous study that used the 3LC exercises among emergency physicians [34]. Previous reports have indicated challenges in integrating the incident command system into healthcare settings due to its bureaucratic framework [51] and the potential for authoritarian leadership [34]. This study, however, demonstrated that the *commands*, *controls*, and *communications* measures could be flexible and adaptive in varied scenarios and could be practically negotiated and compromised through collaboration exercises.

The exercise emphasised the asymmetrical involvements of multiple agencies in various disaster scenarios, ultimately leading to the practical alignment of goals and expectations among the collaborating entities. By exploring different disaster situations, the exercises highlighted the importance of adaptability in effectively managing crises. These findings provided valuable insights into the dynamics of leadership and the effectiveness of various approaches in coordinating multi-agency responses to disasters. Despite the unique challenges and circumstances presented by each disaster scenario, the fundamental principles of disaster management remain grounded in the CSCATTT framework, as supported by existing literature [48, 58]. Thus, the development and implementation of the CSCATTT in response strategies should be prioritised. Moreover, this study focused on analysing cumulative data from all responses to underscore the significance of collaboration and leadership, along with their demonstrated flexibility and resilience.

Regarding *communication*, digital technology has been integrated into daily life from various industries, including healthcare, to daily activities [59, 60], and social media platforms, nowadays, are many people’s primary news sources. Thus, communicating clear information to the public and controlling crowdsourced information is a requirement in emergency management, and the matters were concerned during the simulation [61, 62]. Moreover, the potential social media platforms to distribute updated news to a mass audience was highlighted, as more than 75% of the Thai population consumes the internet [63].

As part of developing *assessment* and *transport* elements, the exercise facilitated an evaluation of the capacities and capabilities of the participants’ organisations, resulting in the development and acknowledgement of surge planning. Community resources were identified, discussed, and integrated into the plan early during the exercise; thus, the FSC was fully implemented. These plans address the knowledge gaps identified in previous reports on transportation flaws and insufficiencies in staff and supplies [4, 24]. Moreover, they are consistent with the UNDRR’s call for all-of-society engagement [10]. Compared to previous studies on community engagement, which often focused on communities’ roles from either the healthcare system’s or people’s perspectives and expectations, this study introduced a more comprehensive and integrated approach [28, 30, 31, 64]. Nevertheless, the exercise did not focus on the patient care process, limiting the potential for improvements in *triage* and *treatment* elements after the training.

Overall, the study demonstrated that the 3LC exercise positively enhanced mutual understanding among participants regarding collaboration, leadership, and individual and organisational flexibility. Participants acknowledged the significance of engaging in collaborative exercises as a mandatory and repetitive component of disaster preparedness and response efforts. The exercise also revealed communication and resource gaps, which are critical factors for effective disaster response and can be addressed to enhance coordination and cooperation, ultimately leading to improved collaboration. This study underscored the need for seamless collaboration among stakeholders, including community resources, for proper disaster preparedness, response, and societal recovery [10, 65, 66]. By fostering collaboration during DPHEs, resources are more efficiently utilized to enhance patient care. It is important to note that while this study engaged communities and disaster response organizations at the district level, future research should consider evaluating the effectiveness of the exercise at regional, national, and international levels. Such research can provide valuable insights for enhancing disaster preparedness and response efforts on a broader scale, considering the complex and interconnected nature of disaster public health emergencies (DPHEs).

### Limitation

The study focused on the impacts of the 3LC exercise on collaborations during the FSC implementation in hospital evacuation events within the Thai context. However, generalising the results to other healthcare systems or communities requires careful analysis of the stakeholders and contexts involved. Regarding leadership performance, it is important to note that due to the utilization of different scenarios in three rounds with seven groups, the sample size was relatively small to differentiate the effects of scenarios or organizations on leadership styles and response efforts. Nonetheless, in practical disaster situations, which are inherently dynamic, high levels of flexibility, adaptability, agility, and resilience are required, and leadership roles and collaboration functions can be interchangeable among organizations. Moreover, the measurements for assessing the impacts were subjectively collected, which may introduce some limitations to the accuracy of the findings. Nevertheless, the study employed a mixed-method design incorporating direct observations of participants’ performances, transcription of the exercise records, and comparative analysis of the findings. Notably, this study represents an initial step towards promoting seamless collaboration among multi-agencies, including communities, in disaster preparedness in Thailand. It could serve as a model for enhancing collaboration among multi-agencies in countries with similar contexts. However, further research on collaboration is necessary to refine plans and conduct full-scale exercises to ensure comprehensive preparedness.

## Conclusion

The 3LC exercise improved participants’ knowledge and understanding of collaboration and leadership during the FSC implementation. Participants were obliged to communicate and align their goals, capacities, and capabilities, ultimately leading to multi-agency coordination, cooperation, and collaboration. Such efforts require continuous training to ensure effective responses, and further research to evaluate its long-term impact could be beneficial. Moreover, engaging community resources demonstrated significant impacts on surge expansion during hospital evacuation preparedness and should be performed as early as possible. This emphasises the importance of involving the community and community members in disaster planning and increasing awareness of resource availability within the disaster response system at a local level.

### Electronic supplementary material

Below is the link to the electronic supplementary material.

Supplementary material 1: The self-evaluation form; Supplementary material 2: The Observational Checklist; Supplementary material 3: The 3LC exercise scenarios

## Data Availability

The datasets generated and analysed during the current study are available in the [Harvard Dataverse] repository, [10.7910/DVN/DNLAEI]

## Abbreviations

3LC: 3 Level Collaboration
COVID-19: Coronavirus 2019
CSCATTT: Command and control, Safety, Communication, Assessment, Triage, Treatment, Transport
DDPM: The Department of Disaster Prevention and Mitigation
DPHEs: Disasters and Public Health Emergencies
FSC: Flexible Surge Capacity
MIMMS: Major Incident Medical Management and Support
UNDRR: The United Nations Office for Disaster Risk Reduction
