# Supplementary material for: The impact of the three-level collaboration exercise on collaboration and leadership during scenario-based hospital evacuation exercises using flexible surge capacity concept: a mixed method cross-sectional study
Source: BMC Health Serv Res. 2023 Aug 14;23:862. doi: 10.1186/s12913-023-09882-x (PMC10426132; doi:10.1186/s12913-023-09882-x)
Supplement: Supplementary file 1 — Supplementary material 1: The self-evaluation form; Supplementary material 2: The Observational Checklist; Supplementary material 3: The 3LC exercise scenarios [file 12913_2023_9882_MOESM1_ESM.docx]

**Supplementary Material 1 The self-evaluation form**

**General information**

Age_______

Genders ☐ Male ☐ Female

**Affiliation**

☐ Provincial Public Health Organization

☐ Hospitals

☐ Provincial Administration

☐ District Administration

☐ Department of Disaster Prevention and Mitigation

☐ Community Please specify ____________ (School, Sports arena, Religious institutes, Head of Community,…)

☐ Police Department

☐ Military

☐ Rescuer/First responder/Non-governmental organization

☐ Others, Please specify _________

**Specific Information**

Please rate your knowledge and understanding in the areas as follow:

|  | Well literacy and can convey the knowledge (5) | Understand and well practice (4) | Average  Understand and practice (3) | Understand but not fluently practice (2) | Know but cannot practice (1) | Never heard of the topic (0) |
| --- | --- | --- | --- | --- | --- | --- |
| Command and Control |  |  |  |  |  |  |
| Safety |  |  |  |  |  |  |
| Communication |  |  |  |  |  |  |
| Assessment |  |  |  |  |  |  |
| Triage |  |  |  |  |  |  |
| Treatment |  |  |  |  |  |  |
| Transport |  |  |  |  |  |  |

Do you think the following organizations associate with disaster response or not? And how?

| Police Departments…………………………………. | Provincial Administrations……………………… |
| --- | --- |
| Fire Departments…………………………………. | Schools…………………………………. |
| Primary Healthcare clinics………………………… | Hotels…………………………………. |
| Private clinics…………………………………. | Sports arenas…………………………………. |
| Dental clinics…………………………………. | Veterinary clinics…………………………………. |
| Others…………………………………. |  |

**Supplementary Material 2 The Observational Checklist**

**Observer checklist: team______________________ Start time: ________________**

|  | Y/N | Who? /What? | Remarks |
| --- | --- | --- | --- |
| **Command and control** | | | |
| Select leader/leadership  If there is no official selection, please state who act as leaders |  |  |  |
| Select other groups members’ tasks and responsibilities |  |  |  |
| Distribute the tasks |  |  |  |
| Use incident command system |  |  |  |
| Recognize the need for collaboration with other organizations |  |  |  |
| Make consensus |  |  |  |
| Resolving the case or each action |  |  |  |
| Outline and establish a multicausalty area including control lines, treatment areas, morgue, transportation areas and vehicle stage areas |  |  |  |
| **Safety** | | | |
| Identify safety officer |  |  |  |
| Identify security team |  |  |  |
| Set safety zoning |  |  |  |
| Control safety area (fire/chemical/infectious) |  |  |  |
| Establish the area for decontamination |  |  |  |
| Monitor safety |  |  |  |
| Recognize what is worth to preserve |  |  |  |
| Recognize personnel protection |  |  |  |
| **Communication** | | | |
| Establish internal communication route |  |  |  |
| Establish inter-agency communication route |  |  |  |
| Establish medical record communication (type and route) |  |  |  |
| Establish backup/alternative system |  |  |  |
| Establish communication protocols/processes |  |  |  |
| Appoint spokesmen (public/media communication) |  |  |  |
| Maintain communication in all means |  |  |  |
| **Assessment** | | | |
| Determine ‘METHANE’ |  |  |  |
| Map structural location (person in charge/access to each zone/function) |  |  |  |
| Maintain assessment/re-assess |  |  |  |
| Surge planning |  |  |  |
| Prioritize mutual resources |  |  |  |
| **Triage** |  |  |  |
| Appoint triage leader |  |  |  |
| Prioritize affected population |  |  |  |
| Treat patients accordingly |  |  |  |
| Consider re-triage |  |  |  |
| **Treatment** |  |  |  |
| Appoint medical treatment leader |  |  |  |
| Medical decision-making |  |  |  |
| Set up of treatment zone |  |  |  |
| **Transport** |  |  |  |
| Appoint transportation unit leader |  |  |  |
| Decision on transport first patient |  |  |  |
| Recognize the need for moving devices |  |  |  |
| Realize the need for safety/protective measures/devices |  |  |  |
| Move the patients to the ambulance transport area |  |  |  |
| Load the patients |  |  |  |
| Surge for alternative transport means |  |  |  |

**Incident command system evaluation**

|  | 5 | 4 | 3 | 2 | 1 | Remarks |
| --- | --- | --- | --- | --- | --- | --- |
| Command | Clear orders and information needed with optimally professional utilized | Clear leader order but not optimal professional utilized | Selection of leader but no clear role | Present of leader but no selection | No lead person |  |
| Control | All follow  All multicausality areas have been organized   1. Gold/silver/bronze or Hot/warm/cold 2. control lines 3. treatment areas 4. morgue 5. transportation areas 6. vehicle stage areas | Most people follow  At least 4 out of 6 areas were organized | Some people follow  Only 2-3 out of 6 areas were organized. | Scarce people follow  Only 1 out of 6  areas were organized. | Not at all  Nothing was organized/appointed |  |
| Safety | Safety officer present with strategies and management plan | Safety officer present with strategies but no visible plan | Safety officer present but no strategies or plan | Safety was considered  Or personal protection was used | Not mention |  |
| Communication  internally | Team member receive information needed and performed correct tasks | Team member receive information needed | Team member receive information needed  No public communication | Internal or external communication were mentioned | No communication was established |  |
| Communication  Externally | - Interagency communication was recognized and collaborated - Public communication was found | - Interagency communication was recognized but independently operated - Public communication was mentioned | Interagency communication and public communication were mentioned | Interagency communication or public communication were mentioned | No communication was established |  |
| Assessment | Scene was thoroughly assessed  (METHANE) | Most of the component were considered | Some of the components were | Some of the components were mentioned | No assessment |  |
| Triage | All patient was correctly triage | Most of patient was correctly triage | Some of patient was correctly triage | Triage was mentioned | No triage |  |
| Treatment | Treatment area was established, and all patient was treated at the area | Treatment area was established, and most patient was treated at the area | Treatment area was established, and come patient was treated at the area | Treatment area was mentioned, or all patient was treated at hospital | No treatment area |  |
| Transport | Transportation modes were surge, optimally utilized, and transport area was organized | Transportation modes were surge with some organized | Transportation was utilized but not organized | Transportation modes were mentioned | No organized transportation mode |  |

**Supplementary Material 3 The 3LC exercise scenarios**

***Scenario 1 flooding with water contamination***

- After a long period of rainfall, a major hospital is flooded, and the water is 1 meter in height. The oxygen tanks and storage are in the basement, which is fully covered by water. There are 10 critical care patients in the ICU, 50 admitted patients in the regular ward, 200 patients in the outpatient departments, and 300 working staff.
- 10 mins later, the safety officer inform the hospital safety team that the water in the supplied tanks is all contaminated with heavy metal and cannot be used.
- HCG decides to evacuate the whole hospital.

***Scenario 2 Pandemic***

- 30 travellers diagnosed with a newly emerging virus are transported directly from the airport to your hospital’s emergency department with respiratory distress condition and need admission. Missing the information, no PPE was used and around 30 emergency department staff, who managed these patients or were in the area, are suspected to have been exposed to the virus. (The ED needs to close for service)
- 5 mins later, all patients are admitted to the in-patient department.
- 10 mins later, you get the information from the provincial public health office that 2 other hospitals have been exposed and contaminated with this newly emerging virus disease.
- There is a shortage of emergency department staff, PPE, ventilators, and negative pressure rooms.

***Scenario 3 Fire and oxygen explosion***

- The inpatient department is on fire. 5 people are injured. The fire is spreading very fast to the three nearest wards. There are 200 patients currently admitted to the hospital and 300 working staff.
- 5 mins later, the fire spreads to the whole floor and extends to other floors.
- 10 mins later, the oxygen tank at the critical care unit explodes.
- A total evacuation is needed.
